# Association between being large for gestational age and cardiovascular metabolic health in children conceived from assisted reproductive technology: a prospective cohort study

**DOI:** 10.1186/s12916-024-03419-7

**Published:** 2024-05-20

**Authors:** Yiyuan Zhang, Kexin Dai, Xiaojing Chen, Linlin Cui, Zi-Jiang Chen

**Affiliations:** 1 Institute of Women, Children and Reproductive Health, Cheeloo College of Medicine, Shandong University, Shandong, 250012 China; 2 Institute of Women, Children and Reproductive Health, the Second Hospital, Cheeloo College of Medicine, Shandong University, Shandong, 250012 China; 3 State Key Laboratory of Reproductive Medicine and Offspring Health, Shandong University, Jinan, Shandong 250012 China; 4 National Research Center for Assisted Reproductive Technology and Reproductive Genetics, Shandong University, Jinan, Shandong 250012 China; 5 Key Laboratory of Reproductive Endocrinology (Shandong University), Ministry of Education, Jinan, Shandong 250012 China; 6 Shandong Technology Innovation Center for Reproductive Health, Jinan, Shandong 250012 China; 7 Shandong Provincial Clinical Research Center for Reproductive Health, Jinan, Shandong 250012 China; 8 Shandong Key Laboratory of Reproductive Medicine, Shandong Provincial Hospital Affiliated to Shandong First Medical University, Jinan, 250012 China; 9 Research Unit of Gametogenesis and Health of ART-Offspring, Chinese Academy of Medical Sciences (No.2021RU001), Jinan, Shandong 250012 China; 10 Department of Reproductive Medicine, Ren Ji Hospital, Shanghai Jiao Tong University School of Medicine, Shanghai, China; 11 Shanghai Key Laboratory for Assisted Reproduction and Reproductive Genetics, Shanghai, China; 12 Jinan, China

**Keywords:** Large for gestational age, Cardiovascular metabolic health, Obesity, Assisted reproductive technology

## Abstract

**Background:**

To the best of our knowledge, no study has investigated the potential joint effect of large for gestational age (LGA) and assisted reproductive technology (ART) on the long-term health of children.

**Methods:**

This was a prospective cohort study that recruited children whose parents had received ART treatment in the Center for Reproductive Medicine, Shandong Provincial Hospital, affiliated to Shandong University, between January 2006 and December 2017. Linear mixed model was used to compare the main outcomes. The mediation model was used to evaluate the intermediary effect of body mass index (BMI).

**Results:**

4138 (29.5%) children born LGA and 9910 (70.5%) children born appropriate for gestational age (AGA) were included in the present study. The offspring ranged from 0.4 to 9.9 years. LGAs conceived through ART were shown to have higher BMI, blood pressure, fasting blood glucose, fasting insulin, and homeostatic model assessment of insulin resistance values, even after controlling for all covariates. The odds of overweight and insulin resistance are also higher in LGA subjects. After adjusting for all covariates, LGAs conceived through ART had BMI and BMI *z*-scores that were 0.48 kg/m^2^ and 0.34 units greater than those of AGAs, respectively. The effect of LGA on BMI was identified as early as infancy and remained consistently significant throughout pre-puberty.

**Conclusions:**

Compared to AGA, LGA children conceived from ART were associated with increased cardiovascular-metabolic events, which appeared as early as infancy and with no recovery by pre-puberty.

**Supplementary Information:**

The online version contains supplementary material available at 10.1186/s12916-024-03419-7.

## Background

The global prevalence of childhood obesity increased by 47.1% between 1980 and 2013 [1], and in 2015, a total of 107.7 million children were classified as obese [2]. Obesity during childhood is a medical disorder that leads to many comorbidities including obstructive sleep apnea syndrome, nonalcoholic fatty liver disease, polycystic ovary syndrome, impaired mobility, joint pain, depression, and anxiety disorders [3]. Furthermore, increasing evidence suggests that obese children are more likely to be classified as obese in adulthood compared to their counterparts with normal weight [4]. Childhood obesity has also been associated with cardiovascular disease and metabolic disorders throughout life [5].

Large for gestational age (LGA), a condition that affects 2.3% to 22.1% of Asian newborns, is generally defined as having a birth weight above the 90^th^ percentile for gestational age [6]. Several factors determine the occurrence of LGA including genes [7], race [8], intrauterine malnutrition [9–11], and artificial intervention in gametes or embryos [12]. Although the long-term health consequences of LGAs were first reported in the 1990s, the condition had not attracted widespread attention until recently. Numerous studies have reported that LGA children (LGAs) have an elevated risk of obesity, hypertension, and metabolic disorders in both childhood and adulthood [13–15]. However, conflicting findings show that a high birth weight is not associated with future cardiovascular and metabolic health problems [16, 17]. Globally, over 8 million children have been conceived through assisted reproductive technology (ART) [18]. However, previous studies demonstrated that children conceived through ART were at a higher risk of developing cardiovascular metabolic challenges in childhood [19, 20]. Nonetheless, to the best of our knowledge, no study has investigated the potential joint effect of LGA and ART on the long-term health of children. Furthermore, little is known about the longitudinal changes associated with LGA, particularly regarding future cardiovascular metabolic health challenges or whether LGAs are able to compensate for the dysfunction over time.

In the present study, we explored the associations between LGA and cardiometabolic markers in the ART cohort. Additionally, we assessed the developmental stages at which the effects of LGA appeared and whether they were compensated for, over time. Furthermore, we examined the intermediary effect of later-life body mass index.

## Methods

### Study design and setting

This study was based on the ART cohort in the Center for Reproductive Medicine, Shandong Provincial Hospital, affiliated to Shandong University, which aimed to evaluate the growth and development of offspring conceived from assisted reproduction technology (ART). It was a prospective cohort that recruited children whose parents received ART treatment at the Center for Reproductive Medicine, Shandong Provincial Hospital Affiliated to Shandong University between January 2006 and December 2017. Recruitment into the study began in July 2014 and was still ongoing at the time of this publication.

The singleton children conceived from ART were eligible for inclusion in the study. However, children were excluded if they had congenital anomalies, kidney or cardiovascular diseases, or lacked anthropometry and cardiovascular metabolism data. In addition, children with a birth weight that was small for gestational age were excluded. Finally, 14,048 children were included in the study (Additional file 1: Fig. S1).

All parents provided signed informed consent and the study was approved by the ethics committee at the Center for Reproductive Medicine, Shandong Provincial Hospital affiliated to Shandong University.

### Exposure assessment

The interest exposure is LGA, which represents a type of “intrauterine overgrowth” [21]. LGA is defined based on three factors: birth weight, gestational age (GA), and sex. Data on birth weight and sex were collected from medical records within 42 days of delivery. Gestational age was calculated using the birth and embryo transfer dates. GA-specific and sex-specific birth weight percentiles based on the Chinese population were used to categorize the GA groups [22]. Those infants exceeding the 90th percentile for a given gestational week are classified as LGA [21]. While infants with birth weight between 10th ~ 90th percentile were considered as appropriate for gestational age (AGA).

### Covariates

Parental demographics, anthropometric data, medical history, and family socioeconomic status were collected before ART treatment. Information on mothers’ exposure to tobacco and alcohol during pregnancy was collected at 13–18 weeks of gestation. Moreover, data on neonatal anthropometry, congenital malformations and diseases, hypertensive disorders in pregnancy (HDP), gestational diabetes mellitus (GDM), and pregnancy anthropometry were collected within 42 days of delivery. The medical history of the parents was defined as whether the parents are diagnosed with hypertension or diabetes, and whether the mother has hyperlipidemia. Parental blood pressure, fasting blood glucose, and maternal lipid profile are routine screening in ART treatment. Exposure to tobacco during pregnancy not only included smoking pregnant women, but also their exposure from the surrounding environment. Family socio-economic status was defined as the parents’ highest socio-economic status. Education level was categorized as three years of college or above and high school or below. Occupation was categorized as student or unemployed, physical labor, and mental labor. Per capita monthly income was categorized as lower than ￥2999, ￥3000 ~ ￥4999, higher than ￥4999. Parity was categorized as first born, second born, and third born or late. The gestational age, children’s age, parents’ age, height, and BMI were adjusted as continuous variables. The categorical covariates such as sex, parity, GDM, HDP, education level, occupation, per capita monthly income, and medical history were converted into a dumbbell variable before adjusted in the models.

### Outcomes

Follow-up visits with children occurred at infancy (0–0.9 years), toddler’s age (1–2.9 years), preschool (3–5.9 years), and school age (6–9.9 years). Children’s projects were determined by the age at visit. The outcomes we focus on include children’s height, BMI, blood pressure (BP), fasting blood glucose (FBG), fasting insulin (FIN), total cholesterol (TC), triacylglycerol (TG), high-density lipoprotein (HDL), and low-density lipoprotein (LDL). We included them as continuous variables and binary variables in the models, respectively.

Height (length) and weight were measured from 5 months. Briefly, height (accurate to 0.1 cm) and weight (accurate to 0.1 kg) were measured twice with a stadiometer and scaled. All children were required to wear light clothes. BMI was calculated as weight/height^2^. The sex-specific BMI *z*-score (*z* = (value-mean)/SD) was calculated based on the mean and the standard deviation (SD) of the 2006 WHO child growth standards. BMI *z*-score > 2 was classified as overweight.

BP was measured from the age of 2 and was estimated three times on the right arm while the child was sitting quietly, using a calibrated electronic BP monitor (Omron HEM-7012, Omron Healthcare, Japan). The mean from the last two BP readings was used for calculation. Sex-, age-, and height-specific BP *z*-score was calculated as (BP-expected BP)/σ. Because the mathematical formula for China’s expected blood pressure and σ cannot be obtained, we used the standard of the National High Blood Pressure Education Program (NHBPEP) [23]. In addition, in order to increase clinical relevancy, we divided blood pressure into SBP or DBP ≥ 95th percentiles of Chinese sex-, age-, and height-specific BP references and < 95th percentiles [24].

Fasting blood samples were obtained from the age of 2. Nurses collected the fasting blood samples in the morning and stored them at – 80 ℃. The hexokinase method (Cobas c702 instrument; Roche Diagnostics, Mannheim, Germany) was used to determine FBG (mmol/L), while the Electrochemiluminescence immunoassay (Cobas e601 instrument; Roche Diagnostics) was utilized to measure serum insulin. Serum TC (mmol/L), TG (mmol/L), LDL (mmol/L), and HDL (mmol/L) were determined using a homogeneous assay (Cobas c702 instrument; Roche Diagnostics). The homeostasis model assessment of insulin resistance (HOMA-IR) was calculated as FIN(mIU/L) × FBG (mmol/L)/22.5. HOMA-IR was divided into ≥ 95th percentiles or < 95th percentiles based on the reference of healthy Chinese children [25]. We divided FBG into two groups based on the diagnostic criteria for impaired fasting glucose tolerance [26]: ≥ 5.6 mmol/L and < 5.6 mmol/L. Similarly, the classification of lipid profiles is also based on the diagnostic criteria for children with hyperlipidemia [27, 28].

### Statistical analysis

Normally distributed data were expressed as means ± SD, skewed variables were presented as the median ± interquartile range, and categorical variables were expressed as numbers and percentages. The *t*-test and Wilcoxon rank sum test were used on continuous variables with normal and skewed distribution, respectively. The chi-square and Fisher’s exact tests were used to analyze categorical data. In addition, the quantile–quantile plot was employed to test for normality.

As this study was designed with repeated measurements, we used the linear mixed model to compare the main linear outcomes. The time-dependent and time-independent variables account for the random effects and fixed effects, respectively. The random effect included the unique offspring ID number. Other covariates were included in the fixed effects. When the outcome is analyzed as a binary variable, the generalized estimating equations (GEE) were applied. The confounding factors are determined through directed acyclic graphs (Additional file 2: Fig. S2). In Model 1, we adjusted for the children’s age and sex, while in Model 2, parity, gestational age, parental age at delivery, HDP, GDM, maternal pre-pregnancy BMI, and maternal tobacco and alcohol exposure during pregnancy, were adjusted. In Model 3, we further adjusted for genetic factors, including parents’ height, BMI, history of hypertension and diabetes, and maternal hyperlipidemia. In Model 4, variables from the above three models were considered along with socioeconomic factors. The number of follow-up visits was additionally adjusted for in each model. Moreover, a generalized additive model allowing for nonlinear correlation was used to describe the age-related trends in BMI, in the two groups.

Notably, a mediation model can separately evaluate the indirect effect of X (independent variable) on Y (dependent variable) through the mediator (M) and its direct effect on Y [29]. BMI was identified as a mediator when assessing the effect of LGA on other outcome variables (metabolic markers). The package of “mediation” was used for mediating effect analysis. Age-stratified analysis was based on developmental stages (toddler’s age: 1–2.9 years old; preschooler: 3–5.9 years; school age children: 6–9.9 years). At each developmental stage, the first follow-up data was used for analysis. The multiple linear models were applied for analysis. Children’s age, sex, parity, gestational age, parental age at delivery, HDP, GDM, maternal tobacco and alcohol exposure during pregnancy, parents’ BMI, parents’ history of hypertension and diabetes, maternal hyperlipidemia, and socioeconomic factors were added to the model.

To account for multiple testing, the Benjamini/Hochberg (B/H) method adjusted two-sided *p* values to control the false discovery rate (FDR). Statistical significance was based on a B/H-adjusted *p* value (*q* value) below 0.05, corresponding to an FDR of 5%. All analyses were conducted in R Version 4.0.2 (R Foundation for Statistical Computing, Vienna, Austria).

## Results

### Participant characteristics

The birth characteristics, parental characteristics, and socio-economic status in included and non-included children were shown in Additional file 3: Tab. S1. Overall, the included and non-included populations have similar birth information, parental characteristics, and socio-economic status. However, the included children tend to have slightly higher birth weight, are more likely to be the first child, have younger parents, and come from families with slightly lower socioeconomic status.

Overall, 9910 (70.5%) children were in the AGA group while 4138 (29.5%) were in the LGA category (Table 1, Additional file 1: Fig. S1). Table 1 gives a summary of the birth, parental, and socioeconomic characteristics of the study participants. The characteristics differed significantly between the two groups. For instance, children in the LGA group had a significantly higher length, birth weight/length (BW/L), birth weight/length^2^ (BW/L^2^), and birth weight/length^3^ (ponder index) (length: mean 51.08 vs. 50.02 cm; BW/L: mean 7.68 vs. 6.56 kg/m; BW/L^2^: mean 15.04 vs. 13.11 kg/m^2^; ponder index: mean 29.50 vs. 26.24 kg/m^3^, all *P* < 0.001). Compared to the AGA category, parents who gave birth to LGA infants were older (age: mother, mean 31.98 vs. 31.40 years; father, mean 32.73 vs. 32.23 years, both *P* < 0.001), higher (height: mother, mean 162.42 vs. 161.43 cm; father, mean 174.18 vs. 173.56 cm, both *P* < 0.001) and heavier (BMI: mother, mean 24.28 vs. 22.87 kg/m^2^; father, mean 25.98 vs. 25.58 kg/m^2^, both *P* < 0.001). Moreover, LGA children were more likely to be exposed to GDM, pre-pregnancy diabetes (GDM: 9.9% vs. 6.4%, pre-pregnancy diabetes: 1.0% vs. 0.2%, all *P* < 0.001).

**Table 1:** Children characteristics, parental characteristics, and family socioeconomic status

	**AGA**	**LGA**	***P*-value**
**Child characteristics at birth**			
**Children, *n***	9910 (70.5%)	4138 (29.5%)	
Female, *n* (%)	5192 (52.4%)	2104 (50.8%)	0.098
Length, cm	50.02 ± 1.66	51.08 ± 2.06	** < 0.001**
Birth weight, g	3280.35 ± 383.29	3921.40 ± 458.58	** < 0.001**
Birth weight/length, kg/m	6.56 ± 0.66	7.68 ± 0.77	** < 0.001**
Birth weight/length^2^, kg/m^2^	13.11 ± 1.25	15.04 ± 1.50	** < 0.001**
Ponder index	26.24 ± 2.60	29.50 ± 3.31	** < 0.001**
Gestational age, weeks	39.11 ± 1.50	38.93 ± 1.58	** < 0.001**
**Maternal characteristics**			
Maternal age at delivery, years	31.40 ± 4.31	31.98 ± 4.38	** < 0.001**
Maternal height, cm	161.43 ± 5.05	162.42 ± 4.87	** < 0.001**
Maternal pre-pregnancy BMI, kg/m^2^	22.87 ± 3.43	24.28 ± 3.69	** < 0.001**
Maternal pre-pregnancy diabetes	20 (0.2%)	40 (1.0%)	** < 0.001**
Gestational diabetes mellitus, *n* (%)	635 (6.4%)	411 (9.9%)	** < 0.001**
Hypertensive disorders in pregnancy, *n* (%)	439 (4.4%)	215 (5.2%)	0.055
Parity, *n* (%)			
First born	8147 (82.2%)	3130 (75.6%)	** < 0.001**
Second born	1700 (17.2%)	968 (23.4%)	
Third born or later	63 (0.6%)	40 (1.0%)	
**Paternal characteristics**			
Paternal age, years	32.23 ± 4.87	32.73 ± 4.94	** < 0.001**
Paternal height, cm	173.56 ± 5.77	174.18 ± 5.85	** < 0.001**
Paternal BMI, kg/m^2^	25.58 ± 4.00	25.98 ± 4.08	** < 0.001**
**Family socioeconomic status**			
Per capita monthly income, *n* (%)			
< 3000 yuan	3421 (34.5%)	1458 (35.2%)	0.112
3000 ~ 4999 yuan	3956 (39.9%)	1620 (39.1%)	
≥ 5000 yuan	2151 (21.7%)	868 (21.0%)	
Highest occupation			
Student or unemployed	150 (1.5%)	63 (1.5%)	**0.002**
Physical labor	6517 (65.8%)	2857 (69.0%)	
Mental labor	3243 (32.7%)	1217 (29.4%)	
Highest education			
^a^University or above	4500 (45.4%)	1703 (41.2%)	** < 0.001**
^b^High school or below	5410 (54.6%)	2434 (58.8%)	

Data presented as mean ± SD for continuous variables and *n* (%) for categorical variables

*Abbreviation*: *BMI* body mass index

^a^University or above: junior college, undergraduate, master’s, and doctoral students

^b^High school or below: illiteracy, primary school, junior high school, and high school

### Anthropometric measures and cardiometabolic makers

There were striking anthropometric differences between children in the LGA and AGA groups (Tables 2 and 3, Additional file 4: Tab. S2). Compared with AGA subjects, LGAs conceived from ART subjects had significantly higher odds of overweight (odds ratio (OR) = 1.59, 95% confidence interval (CI): 1.46–1.74; Table 2). LGAs conceived from ART had BMI and BMI *z*-scores that were 0.48 kg/m^2^ and 0.34 units greater than those of AGAs, respectively. Additionally, LGA children were about 1.09 cm taller in height and had 0.39 more height *z*-score units compared to those in the AGA group (height: mean 88.17 vs. 86.43 cm, height *z*-score: mean 1.10 vs. 0.65). At birth, length and Ponder index were higher in LGA newborns than in their AGA peers (both *P* < 0.001) (Table 1). Moreover, higher BMI and BMI *z*-scores were observed in the LGA group from infancy to pre-puberty, and after adjusting for confounders, the difference was still significant (BMI kg/m^2^: infancy, mean 18.68 vs. 18.13; toddler’s age, mean 16.94 vs. 16.44; preschooler, mean 16.44 vs. 15.83; school age children, mean 17.96 vs. 16.99; BMI *z*-score: infancy, mean 1.07 vs. 0.70, toddler’s age, mean 0.70 vs. 0.33, preschooler, mean 0.78 vs. 0.34, school age children, mean 1.37 vs. 0.79) (Table 4). The significantly higher BMI, BMI *z*-score, height, height *z*-score were observed in both male and female LGA children conceived through ART (Additional file 5: Tab. S3, Additional file 6: Fig. S3). Similarly, the significantly higher BMI, BMI *z*-score, height, height *z*-score were observed both in fresh embryo transfer and frozen embryo transfer (Additional file 7: Tab. S4).

**Table 2:** ORs and 95% CIs for the association between LGA and adverse levels of CVD risk factors

	AGA	LGA	Unadjusted		Adjusted	
			OR (95% CI)	*q* value	OR (95% CI)	*q* value
BMI *z*-score > 2	1467 (9.4%)	1025 (15.5%)	**1.78 (1.63, 1.94)**	** < 0.001**	**1.59 (1.46, 1.74)**	** < 0.001**
BP^a^ ≥ 95th percentile	680 (18.5%)	346 (21.0%)	1.14 (0.99, 1.31)	0.140	1.12 (0.97, 1.29)	0.220
FBG ≥ 5.6 mmol/L	173 (4.4%)	103 (5.9%)	**1.35 (1.07, 1.70)**	**0.032**	1.35 (1.05, 1.74)	0.053
HOMA-IR ≥ 95th percentile	156 (4.0%)	116 (6.7%)	**1.66 (1.33, 2.08)**	** ≤ 0.001**	**1.58 (1.22, 2.06)**	**0.002**
TC ≥ 5.17 mmol/L	265 (6.7%)	113 (6.5%)	0.96 (0.77, 1.20)	0.740	0.97 (0.77, 1.22)	0.882
TG ≥ 1.12 mmol/L	443 (11.2%)	203 (11.7%)	1.04 (0.88, 1.23)	0.740	1.01 (0.85, 1.21)	0.882
LDL ≥ 3.36 mmol/L	261 (6.6%)	126 (7.2%)	1.10 (0.88, 1.37)	0.624	1.11 (0.89, 1.39)	0.552
HDL ≤ 1.03 mmol/L	345 (8.8%)	145 (8.3%)	0.95 (0.78, 1.15)	0.740	0.92 (0.75, 1.12)	0.552

Bolded variables indicate statistical significance (*q* ≤ 0.05)

Adjusted for children’s age and sex, parity, gestational age, parental age at delivery, HDP, GDM, maternal tobacco and alcohol exposure during pregnancy, parents’ BMI, parents’ history of hypertension and diabetes, maternal hyperlipidemia, socioeconomic factors

*Abbreviations*: *BMI* body mass index, *BP* blood pressure, *FBG* fasting blood glucose, *FIN* fasting insulin, *HOMA-IR* homeostatic model assessment for insulin resistance, *LDL* low-density lipoprotein, *HDL* high-density lipoprotein

^a^According to the American Academy of Pediatrics diagnostic criteria for hypertension, only children ≥ 3 years old are included

**Table 3:** Unadjusted and measures of anthropometry, metabolic markers of offspring born AGA, or LGA

	AGA	LGA	Unadjusted		Adjusted	
			SE	*q* value	SE	*q* value
**Anthropometric characteristics**						
*n*	15,739	6649				
Age, years	2.12 ± 1.84	2.17 ± 1.89				
BMI**,** kg/m^2^	16.89 ± 2.01	17.46 ± 2.14	**0.04**	** < 0.001**	**0.03**	** < 0.001**
BMI *z*-score	0.47 ± 1.22	0.88 ± 1.30	**0.02**	** < 0.001**	**0.02**	** < 0.001**
^a^Height, cm	86.43 ± 16.97	88.17 ± 17.52	**0.30**	** < 0.001**	**0.07**	** < 0.001**
^a^Height *z*-score	0.65 ± 1.06	1.10 ± 1.07	**0.02**	** < 0.001**	**0.02**	** < 0.001**
**BP**						
*n*	6639	2937				
Age, years	3.69 ± 1.80	3.72 ± 1.82				
SBP, mmHg	93.40 ± 8.93	94.14 ± 9.23	**0.21**	**0.002**	**0.18**	**0.002**
SBP *z*-score	0.01 ± 0.76	0.00 ± 0.90	0.02	0.555	0.02	0.614
DBP, mmHg	56.76 ± 9.41	57.76 ± 9.64	**0.23**	** < 0.001**	**0.20**	** < 0.001**
DBP *z*-score	0.59 ± 0.76	0.62 ± 0.88	0.02	0.067	**0.02**	**0.004**
**Metabolic characteristics**						
***n***	3951	1751				
Age, years	4.63 ± 1.80	4.67 ± 1.81				
FBG, mmol/L	4.94 ± 0.42	4.98 ± 0.43	**0.01**	**0.002**	**0.01**	**0.009**
FIN, mIU/L	5.12 ± 3.90	5.61 ± 4.68	**0.13**	**0.001**	**0.12**	**0.043**
HOMA-IR	1.15 ± 0.94	1.29 ± 1.19	**0.03**	** < 0.001**	**0.03**	**0.017**
TC, mmol/L	4.03 ± 0.71	4.07 ± 0.70	0.02	0.287	0.02	0.170
TG, mmol/L	0.76 ± 0.33	0.76 ± 0.30	0.01	0.981	0.01	0.721
LDL, mmol/L	2.41 ± 0.59	2.45 ± 0.59	0.02	0.238	0.02	0.102
HDL, mmol/L	1.41 ± 0.30	1.41 ± 0.29	0.01	0.981	0.01	0.896

Data presented as mean ± SD for continuous variables and *n* (%) for categorical variables

Bolded variables indicate statistical significance (*q* ≤ 0.05)

Adjusted for children’s age and sex, parity, gestational age, parental age at delivery, HDP, GDM, maternal tobacco and alcohol exposure during pregnancy, parents’ BMI, parents’ history of hypertension and diabetes, maternal hyperlipidemia, socioeconomic factors

*Abbreviations*: *SE* standard error, *BMI* body mass index, *BP* blood pressure, *SBP* systolic blood pressure, *DBP* diastolic blood pressure, *FBG* fasting blood glucose, *FIN* fasting insulin, *HOMA-IR* homeostatic model assessment for insulin resistance, *LDL* low-density lipoprotein, *HDL* high-density lipoprotein

^a^Adjusted for children’s age and sex, parity, gestational age, parental age at delivery, HDP, GDM, maternal tobacco and alcohol exposure during pregnancy, parents’ height, parents’ history of hypertension and diabetes, maternal hyperlipidemia, socioeconomic factors

**Table 4:** Measures of anthropometry, metabolic markers of offspring born AGA, or LGA which stratified by age

	AGA	LGA	Unadjusted		Adjusted	
			SE	*q* value	SE	*q* value
**Infancy**	4958	2100				
Age, years	0.55 ± 0.13	0.55 ± 0.13				
BMI, kg/m^2^	18.13 ± 1.79	18.68 ± 1.86	**0.05**	** < 0.001**	**0.05**	** < 0.001**
BMI *z*-score	0.70 ± 1.19	1.07 ± 1.23	**0.03**	** < 0.001**	**0.03**	** < 0.001**
Height, cm^b^	69.68 ± 3.38	70.72 ± 3.19	**0.09**	** < 0.001**	**0.06**	** < 0.001**
Height *z*-score^b^	0.92 ± 1.11	1.44 ± 1.06	**0.03**	** < 0.001**	**0.03**	** < 0.001**
**Toddler’s age**^**a**^	7040	2875				
Age, years	1.81 ± 0.62	1.82 ± 0.62				
BMI, kg/m^2^	16.44 ± 1.64	16.94 ± 1.67	**0.04**	** < 0.001**	**0.04**	** < 0.001**
BMI *z*-score	0.33 ± 1.12	0.70 ± 1.13	**0.03**	** < 0.001**	**0.03**	** < 0.001**
Height, cm^b^	85.67 ± 7.37	87.13 ± 7.39	**0.16**	** < 0.001**	**0.07**	** < 0.001**
Height *z*-score^b^	0.54 ± 1.03	0.99 ± 1.03	**0.03**	** < 0.001**	**0.02**	** < 0.001**
SBP, mmHg	89.40 ± 6.11	89.86 ± 6.42	0.21	0.065	**0.20**	**0.014**
SBP *z* score	− 0.19 ± 0.56	− 0.20 ± 0.60	0.02	0.504	0.02	0.523
DBP, mmHg	53.38 ± 7.73	53.96 ± 8.02	0.26	0.065	**0.27**	**0.009**
DBP *z* score	0.62 ± 0.69	0.64 ± 0.72	0.02	0.504	0.02	0.266
FBG, mmol/L	4.84 ± 0.45	4.83 ± 0.45	0.04	0.915	0.04	0.695
FIN, mIU/L	3.90 ± 3.04	3.58 ± 2.65	0.23	0.381	0.24	0.260
HOMA-IR	0.88 ± 0.89	0.80 ± 0.69	0.07	0.504	0.07	0.364
TC, mmol/L	4.02 ± 0.70	4.01 ± 0.69	0.06	0.985	0.06	0.790
TG, mmol/L	0.70 ± 0.28	0.70 ± 0.31	0.02	0.998	0.02	0.903
LDL, mmol/L	2.39 ± 0.59	2.39 ± 0.60	0.05	0.998	0.05	0.903
HDL, mmol/L	1.42 ± 0.30	1.42 ± 0.28	0.02	0.998	0.02	0.903
**Preschooler**	2823	1237				
Age, years	3.92 ± 0.88	3.92 ± 0.89				
BMI, kg/m^2^	15.83 ± 1.72	16.44 ± 1.80	**0.06**	** < 0.001**	**0.06**	** < 0.001**
BMI *z*-score	0.34 ± 1.26	0.78 ± 1.31	**0.05**	** < 0.001**	**0.05**	** < 0.001**
Height, cm^b^	103.82 ± 7.75	105.31 ± 7.83	**0.27**	** < 0.001**	**0.14**	** < 0.001**
Height *z*-score^b^	0.40 ± 0.96	0.77 ± 1.04	**0.04**	** < 0.001**	**0.03**	** < 0.001**
SBP, mmHg	94.94 ± 9.13	95.70 ± 9.25	**0.32**	**0.032**	**0.31**	**0.047**
SBP *z* score	0.15 ± 0.81	0.12 ± 1.11	0.03	0.515	0.03	0.703
DBP, mmHg	57.95 ± 9.86	59.18 ± 9.91	**0.35**	**0.001**	**0.35**	**0.001**
DBP *z* score	0.58 ± 0.85	0.63 ± 1.09	0.03	0.203	**0.02**	**0.041**
FBG, mmol/L	4.91 ± 0.43	4.95 ± 0.42	**0.02**	**0.015**	0.02	**0.017**
FIN, mIU/L	4.38 ± 2.89	4.73 ± 3.25	**0.11**	**0.003**	0.11	0.081
HOMA-IR	0.98 ± 0.69	1.07 ± 0.81	**0.03**	**0.002**	0.03	**0.041**
TC, mmol/L	4.06 ± 0.73	4.10 ± 0.70	0.03	0.129	0.03	0.115
TG, mmol/L	0.76 ± 0.30	0.76 ± 0.30	0.01	0.901	0.01	0.692
LDL, mmol/L	2.44 ± 0.60	2.48 ± 0.57	0.02	0.102	0.02	0.098
HDL, mmol/L	1.39 ± 0.30	1.38 ± 0.28	0.01	0.907	0.01	0.803
**School age**	918	437				
Age, years	7.40 ± 1.00	7.35 ± 0.91				
BMI, kg/m^2^	16.99 ± 3.03	17.96 ± 3.68	**0.20**	** < 0.001**	**0.20**	**0.033**
BMI *z*-score	0.79 ± 1.67	1.37 ± 2.08	**0.12**	** < 0.001**	**0.12**	**0.023**
Height, cm^b^	128.29 ± 8.45	129.68 ± 7.83	**0.50**	**0.008**	**0.34**	** < 0.001**
Height *z*-score^b^	0.86 ± 0.99	1.17 ± 1.02	**0.06**	** < 0.001**	**0.06**	** < 0.001**
SBP, mmHg	101.72 ± 8.84	102.53 ± 9.16	0.54	0.263	0.55	0.835
SBP *z*-score	0.23 ± 0.80	0.25 ± 0.83	0.05	0.762	0.05	0.481
DBP, mmHg	64.16 ± 7.76	65.12 ± 7.85	0.47	0.116	0.49	0.154
DBP *z*-score	0.47 ± 0.67	0.52 ± 0.67	0.04	0.332	0.04	0.465
FBG, mmol/L	5.07 ± 0.37	5.15 ± 0.37	**0.02**	**0.001**	**0.02**	**0.001**
FIN, mIU/L	7.84 ± 5.25	8.83 ± 6.57	**0.35**	**0.014**	**0.37**	0.076
HOMA-R	1.79 ± 1.23	2.06 ± 1.71	**0.09**	**0.008**	**0.09**	**0.033**
TC, mmol/L	3.97 ± 0.66	4.03 ± 0.71	0.04	0.381	0.05	0.180
TG, mmol/L	0.79 ± 0.41	0.78 ± 0.32	0.02	0.734	0.02	0.656
LDL, mmol/L	2.35 ± 0.57	2.40 ± 0.61	0.04	0.359	0.04	0.134
HDL, mmol/L	1.47 ± 0.31	1.48 ± 0.32	0.02	0.971	0.02	0.835

Data presented as mean ± SD for continuous variables and *n* (%) for categorical variables

Bolded variables indicate statistical significance (*q* ≤ 0.05)

Infancy: 0.5–0.9 years old; toddler’s age: 1–2.9 years old; preschooler: 3–5.9 years; **s**chool age children: 6–9.9 years

Adjusted for children’s age and sex, parity, gestational age, parental age at delivery, HDP, GDM, weight gain during pregnancy, maternal tobacco and alcohol exposure during pregnancy, parents’ BMI, parents’ history of hypertension and diabetes, maternal hyperlipidemia, socioeconomic factors

*Abbreviations*: *SE* standard error, *BMI* body mass index, *SBP* systolic blood pressure, *DBP* diastolic blood pressure, *FBG* fasting blood glucose, *FIN* fasting insulin, *HOMA-IR* homeostatic model assessment for insulin resistance, *LDL* low-density lipoprotein, *HDL* high-density lipoprotein

^a^For blood pressure, the number of AGA and LGA groups were 2960 and 1291, respectively; for metabolic markers, the number of AGA and LGA groups were 500 and 226, respectively

^b^Adjusted for children’s age and sex, parity, gestational age, parental age at delivery, HDP, GDM, weight gain during pregnancy, maternal tobacco and alcohol exposure during pregnancy, parents’ height, parents’ history of hypertension and diabetes, maternal hyperlipidemia, socioeconomic factors

LGAs conceived through ART had significantly higher systolic blood pressure (SBP) and diastolic blood pressure (DBP) values than the AGAs (SBP: mean 94.14 vs. 93.40 mmHg; DBP: mean 57.76 vs. 56.76) (Table 3, Additional file 4: Tab. S2). In addition, age-specific analysis revealed that LGAs conceived through ART were associated with a significantly higher BP at toddler’s age and pre-school age but not in school age subgroup children (SBP: toddler’s age, mean 89.86 vs. 89.40 mmHg, *q* = 0.014; preschooler, mean 95.70 vs. 94.94 mmHg, *q* = 0.047; school age children, mean 102.53 vs. 101.72 mmHg, *q* = 0.835; DBP: toddler’s age, mean 53.96 vs. 53.38 mmHg, *q* = 0.009; preschooler, mean 59.18 vs. 57.95 mmHg, *q* = 0.001; school age children, mean 65.12 vs. 64.16 mmHg, *q* = 0.154) (Table 4).

Compared with AGA subjects, LGAs conceived from ART subjects had significantly higher odds of insulin resistance (greater than or equal 95th percentile of HOMA-IR of healthy Chinese children) (OR = 1.58, 95% CI: 1.22–2.06; Table 2). FBG, FIN, and HOMA-IR were significantly higher in LGA children conceived through ART than in their AGA counterparts (FBG: mean 4.98 vs. 4.94 mmol/L; FIN: mean 5.61 vs. 5.12 mIU/L; HOMA-IR: mean 1.29 vs. 1.15) (Table 3). However, there were no significant differences in TC, TG, LDL, and HDL, between the LGA and AGA groups. When stratified by age, there were no significant differences in FBG, FIN, and HOMA-IR between LGA and AGA toddlers (Table 4). In addition, FBG and HOMA-IR were significantly higher in the LGA group, among preschool age and school age children (Table 4). Stratification based on gender yielded different results. FBG was significantly higher in the LGA group, among females (FBG: mean 4.94 vs. 4.89 mmol/L, FIN: mean 5.80 vs. 5.27 mIU/L; HOMA-IR: mean 1.32 vs. 1.17) (Additional file 5: Tab. S3). However, there were no differences in FBG, FIN, and HOMA-IR between the LGA and AGA groups, in males (Additional file 5: Tab. S3). Additionally, in male subjects, TC and LDL levels were significantly higher in the LGA group, even after correcting for confounders (Additional file 5: Tab. S3).

### Mediating

The direct and indirect effects of LGA are presented in Table 5. For FBG, FIN, and HOMA-IR the mediating effect of BMI is significant from preschool age to school age children. And in toddler’s age, the mediating effect of BMI for FBG and FIN is also significant (although it no longer has statistical significance after adjusting the *p*). Higher BMI in LGA children may have adverse effects on glucose metabolism at all developmental stages we have studied. However, the total effect a significant only in school age children (Because the mediation effect analysis only included the first follow-up data for each developmental stage, the results were slightly different from the results in Table 4). In school age children, the mediating effects of BMI on FIN and HOMA-IR accounted for 52.4% and 42.9% respectively, which indicated that when the elevated BMI cannot return to normal, LGA children may have adverse glucose metabolism outcomes in the future. For SBP and DBP the mediating effect of BMI is significant at all developmental stages we have studied. In other words, elevated BMI can have adverse effects on BP, regardless of developmental stage.

**Table 5:** Estimate coefficients (95% CI) of measures of anthropometry and metabolic markers according to LGA

	Indirect effect	Direct effects	Total effect	Percentage
**Toddler’s age**				
SBP, mmHg	**0.21 (0.14, 0.28)**	0.36 (− 0.06, 0.74)	**0.56 (0.16, 0.97)**	35.4%
DBP, mmHg	**0.26 (0.17, 0.35)**	0.56 (0.07, 1.05)	**0.82 (0.30, 1.32)**	31.7%
FBG, mmol/L	0.01 (0, 0.03)	− 0.03 (− 0.10, 0.04)	− 0.02 (− 0.09, 0.05)	18.0%
FIN, mIU/L	0.09 (0, 0.18)	− 0.45 (− 0.92, 0.01)	− 0.36 (− 0.85, 0.09)	21.0%
HOMA-IR	0.02 (0, 0.05)	− 0.11 (− 0.25, 0.04)	− 0.09 (− 0.23, 0.06)	17.5%
**Preschooler**				
SBP, mmHg	**0.48 (0.34, 0.61)**	0.19 (− 0.45, 0.80)	0.67 (0.05, 1.28)	70.3%
DBP, mmHg	**0.37 (0.24, 0.52)**	**1.15 (0.42, 1.85)**	**1.51 (0.79, 2.24)**	24.2%
FBG, mmol/L	**0.01 (0.01, 0.02)**	0.02 (− 0.01, 0.05)	0.03 (0, 0.07)	34.0%
FIN, mIU/L	**0.25 (0.17, 0.33)**	− 0.13 (− 0.33, 0.08)	0.12 (− 0.08, 0.34)	100%
HOMA-IR	**0.06 (0.04, 0.08)**	− 0.02 (− 0.07, 0.03)	0.04 (− 0.01, 0.09)	100%
**School age**				
SBP, mmHg	**0.53 (0.11, 0.98)**	− 0.66 (− 1.73, 0.42)	− 0.13 (− 1.30, 1.01)	38.4%
DBP, mmHg	**0.38 (0.06, 0.72)**	0.33 (− 0.66, 1.29)	0.71 (− 0.33, 1.74)	44.0%
FBG, mmol/L	**0.01 (0, 0.01)**	**0.08 (0.03, 0.13)**	**0.09 (0.04, 0.14)**	6%
FIN, mIU/L	**0.42 (0.11, 0.75)**	0.38 (− 0.23, 0.95)	**0.80 (0.16, 1.46)**	52.4%
HOMA-IR	**0.10 (0.02, 0.19)**	0.14 (− 0.02, 0.31)	**0.24 (0.07, 0.41)**	42.9%

Percentage: percentage of mediating effect in total effect

Adjusted for children’s age and sex, parity, gestational age, parental age at delivery, HDP, GDM, maternal tobacco and alcohol exposure during pregnancy, parents’ BMI, parents’ history of hypertension and diabetes, maternal hyperlipidemia, socioeconomic factors

Bolded variables indicate statistical significance (*q* ≤ 0.05)

*Abbreviations*: *SBP* systolic blood pressure, *DBP* diastolic blood pressure, *FBG* fasting blood glucose, *FIN* fasting insulin, *HOMA-IR* homeostatic model assessment for insulin resistance

## Discussion

In the present study, involving children aged 0.4–9.9 years, LGAs conceived through ART were shown to have higher cardiovascular risk profiles than their AGA counterparts, including higher BMI, BP, FBG, FIN, and HOMA-IR values even after controlling for all covariates. The odds of overweight and insulin resistance are also higher in LGA subjects. The elevated BMI appeared as early as in infancy in the LGA children and remained consistently high to their pre-puberty.

Consistent with previous research, our study showed that LGA individuals had a higher BMI [15, 30–32]. The determinants of LGA including GDM and parental obesity have been confirmed to be independent risk factors for childhood obesity [33, 34]. Even after adjusting for those covariates, LGAs still had a significantly higher BMI which was consistent with earlier studies that demonstrated the independent effect of LGA [16, 30, 32]. However, several studies did not show the influence of LGA in children born to women without GDM and obesity [13, 35]. But the positive association between the LGA and BMI was found in our sensitivity analysis which excluded those children whose mothers suffer from GDM and obesity (Additional file 8: Tab. S5). Although there is limited data available to explain this effect, one potential biological mechanism is “fetal programming.” Caused by an adverse early life environment, “fetal programming” could influence the development of the liver and hypothalamus, affect the volume, number, and distribution of adipocytes, alter gene expression levels, and change responses to the postnatal nutritional environment [36–38].

This study also showed that the ponderal index of LGA newborns was significantly higher than that of AGA newborns. Similarly, previous studies on the body composition of newborns demonstrated that LGA children born to women with GDM and obesity had an increase in both fat and lean body mass [39]. Moreover, our study also indicated that the elevated BMI in LGAs appeared as early as in infancy and remained consistent to pre-puberty. And the studies focused on adolescence and adulthood also found a high risk of obesity in LGAs [14, 40]. Evidence of childhood obesity persisting into adulthood was confirmed in a systematic review including 25 publications [4]. Therefore, LGA is not only a marker of “fetal obesity” but is also the earliest indicator of future obesity. According to previous research, an effort to normalize BMI before puberty was beneficial as it was shown to significantly reduce the risk of metabolic disorders and cardiovascular disease in adulthood [41, 42]. Furthermore, existing literature shows that if obesity starts at around the age of 7 and BMI increases from age 7 to adolescence, the risk of type 2 diabetes in middle age increases significantly, even if the weight was normal before age 7 [41]. However, the BMI gap between the LGAs and AGAs did not disappear with age in both genders, which might indicate that LGA was not “metabolically normal obese.”

In the present study, higher odds of insulin resistance and higher FBG, FIN, and HOMA-IR values were observed in LGA children compared to their AGA peers, even after adjusting for covariates. These results were consistent with those previous studies, which showed that LGA was positively associated with increased insulin resistance [13, 35, 43]. However, contrary to our findings, numerous previous studies did not report significant differences in glucose metabolism between the LGAs and AGAs [16, 44, 45]. The difference in BMI between LGA and AGA children may be the reason for the contradictory results. According to existing reports, BMI was significantly correlated with insulin resistance, increased BMI occurred earlier than insulin resistance, and BMI interventions could significantly improve insulin sensitivity [46–48]. Our intermediary effect analysis showed that the impact of LGA on children’s FIN and HOMRA-IR was explained by elevated BMI. The results also showed that impaired glucose metabolism in LGA children was more significant in girls. The potential underlying mechanisms include differential expression of metabolic-related genes, varying hormone sensitivity, and body composition [49, 50]. The different sex ratios in previous studies may also be the reason for the conflicting results. Moreover, the heterogeneity of the population might be a potential reason. In this study, significant differences in BMI and glucose metabolism were observed in children conceived through ART. However, it was not clear if there was any interaction between ART and LGA.

In the present study, higher blood pressure was observed in LGA children compared to their AGA peers. Moreover, the age subgroup showed that a higher BP in LGA children was observed in toddlers and preschoolers, but not in school aged children. Previous studies suggested that being LGA was associated with a significantly higher risk of hypertension by 1.4–1.8-fold in adolescents and young adults, but not in school aged or younger children [51, 52]. However, other reports did not show significant differences between LGAs and AGAs in either SBP or DBP [17, 35, 53]. The intermediary effect analysis showed that elevated BMI could explain approximately one-fourth to two-thirds of the effect of LGA on BP. The difference in BMI could be one of the reasons for the inconsistent results. A previous study reported that children conceived through ART were associated with an increased risk of cardiovascular metabolic dysfunction, compared to the natural controls [19]. Another possible explanation might be the interaction between LGA and ART.

This study had several strengths including the use of longitudinal and detailed data, a wide range of covariates, a homogeneous population, and a large sample size. However, despite the insightful findings, it also had some limitations. First, although various measures were taken to enhance the response rate, non-response was inevitable (Additional file 1: Fig. S1, Additional file 9: Tab. S6). Children with younger parents are more likely to receive follow-up (Additional file 3: Tab. S1), which may lead to selection bias.

In addition, despite our best efforts to adjust for confounding factors, we cannot observe all confounding factors (known and unknown). Secondly, based on current data, we are unable to distinguish LGA represents a condition arising from physiological processes and pathological conditions. Our analysis represents the combined results of all types of LGA, and the more detailed analysis requires further well-designed research [54]. In addition, live birth is one of the necessary inclusion criteria for evaluating the long-term health of offspring born from ART, which inevitably leads to selection bias. This study is more representative of the children born to couples who are able to have live birth infants. And this study is focused on participants from the ART cohort, whether the results can be extrapolated to the naturally pregnant population requires more well-designed research to confirm. Third, the samples were detected on different dates because of the large sample size. Our biospecimen results may be influenced by the potential batch effects.

## Conclusions

In conclusion, LGA conceived from ART were at a higher risk of cardiovascular metabolic dysfunction in childhood, were more predisposed to obesity, and had decreased insulin sensitivity. These could be identified as early as in infancy and persisted consistently to pre-puberty. In addition, the BMI in LGA children may be an intermediary that serves to block further deterioration of cardiovascular metabolism. Therefore, controlling birth weight and post birth overweight and obesity may be effective measures to reduce cardiovascular metabolic risks in children conceived from ART. And prevention of overweight and obesity in these children should begin at infancy.

### Supplementary Information

Additional file 1: Fig. S1. Flow Chart.

Additional file 2: Fig. S2. Direct Acyclic Graph.

Additional file 3: Tab. S1. Children Characteristics, Parental Characteristics, and Family Socioeconomic Status in Included and Non-included Singleton Children Conceived from ART.

Additional file 4: Tab. S2. Effect of LGA on Measures of Childhood Anthropometric Data and Metabolisms.

Additional file 5: Tab. S3. Measures of Anthropometry, Metabolic Markers of Offspring Born AGA, or LGA which Stratified by Children’s Gender.

Additional file 6: Fig. S3. Visualization of BMI Changes with Age.

Additional file 7: Tab. S4. Measures of Anthropometry, Metabolic Markers of Offspring Born AGA, or LGA which Stratified by whether Embryos are Frozen.

Additional file 8: Tab. S5. Measures of Anthropometry, Metabolic Markers of Offspring Born AGA, or LGA in Children Whose Mother without Obesity, Gestational Diabetes Mellitus, and Hyperlipidemia.

Additional file 9: Tab. S6. Number of Children at Each Follow-up Stage.

## Data Availability

The datasets used and/or analyzed during the current study are available from the corresponding author on reasonable request.

## Abbreviations

AGA: Appropriate for gestational age
ART: Assisted reproductive technology
B/H: Benjamini/Hochberg
BMI: Body mass index
BP: Blood pressure
BW/L: Birth weight/length
BW/L^2^: Birth weight/length^2^
CI: Confidence interval
DBP: Diastolic blood pressure
FBG: Fasting blood glucose
FDR: False discovery rate
FIN: Fasting insulin
GA: Gestational age
GDM: Gestational diabetes mellitus
GEE: Generalized estimating equations
HDL: High-density lipoprotein
HDP: Hypertensive disorders in pregnancy
HOMA-IR: Homeostatic model assessment of insulin resistance
LDL: Low-density lipoprotein
LGA: Large for gestational age
NHBPEP: National High Blood Pressure Education Program
OR: Odds ratio
SBP: Systolic blood pressure
SD: Standard deviation
TC: Total cholesterol
TG: Triacylglycerol
