# Supplementary figures and images for: Association between being large for gestational age and cardiovascular metabolic health in children conceived from assisted reproductive technology: a prospective cohort study
Source: BMC Med. 2024 May 20;22:203. doi: 10.1186/s12916-024-03419-7 (PMC11104001; doi:10.1186/s12916-024-03419-7)

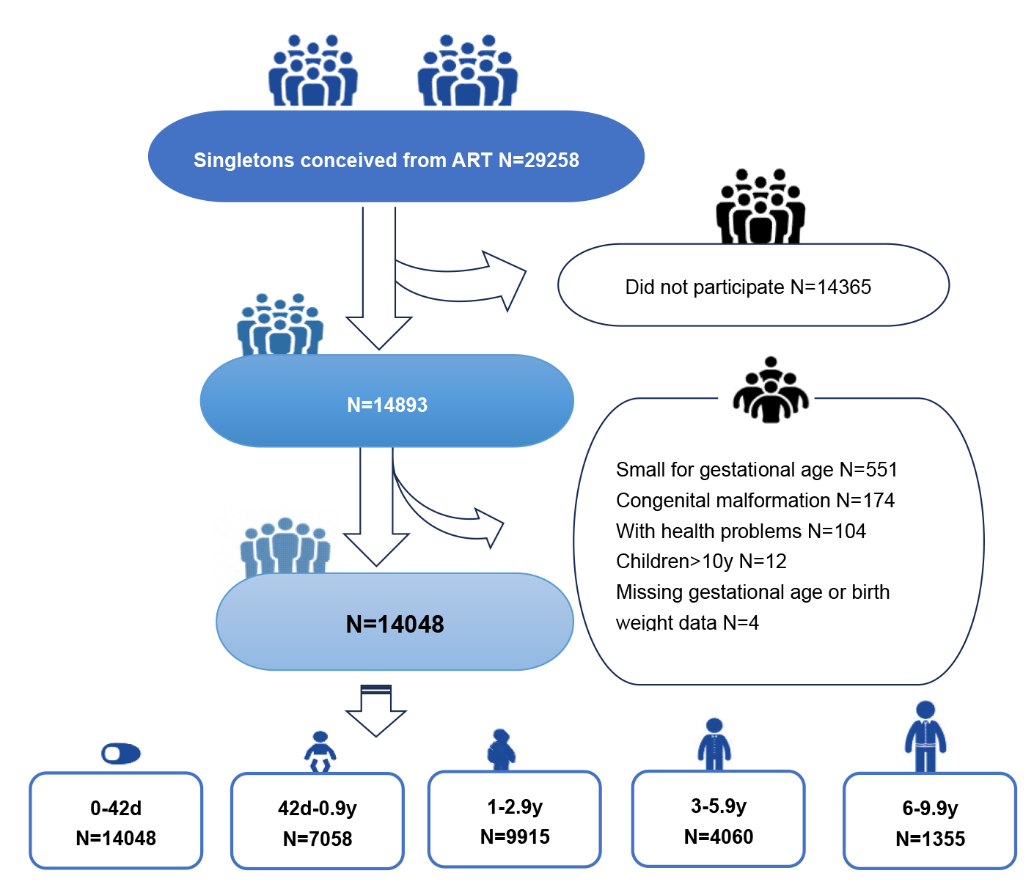


**Supplementary Figure 1:** Flow Chart

**Abbreviation:** ART, Assisted reproductive technology.

Supplement: Supplementary file 1 — Additional file 1: Fig. S1. Flow Chart. [file 12916_2024_3419_MOESM1_ESM.docx]
