# Supplementary material for: Association between being large for gestational age and cardiovascular metabolic health in children conceived from assisted reproductive technology: a prospective cohort study
Source: BMC Med. 2024 May 20;22:203. doi: 10.1186/s12916-024-03419-7 (PMC11104001; doi:10.1186/s12916-024-03419-7)
Supplement: Supplementary file 2 — Additional file 2: Fig. S2. Direct Acyclic Graph. [file 12916_2024_3419_MOESM2_ESM.docx]

**
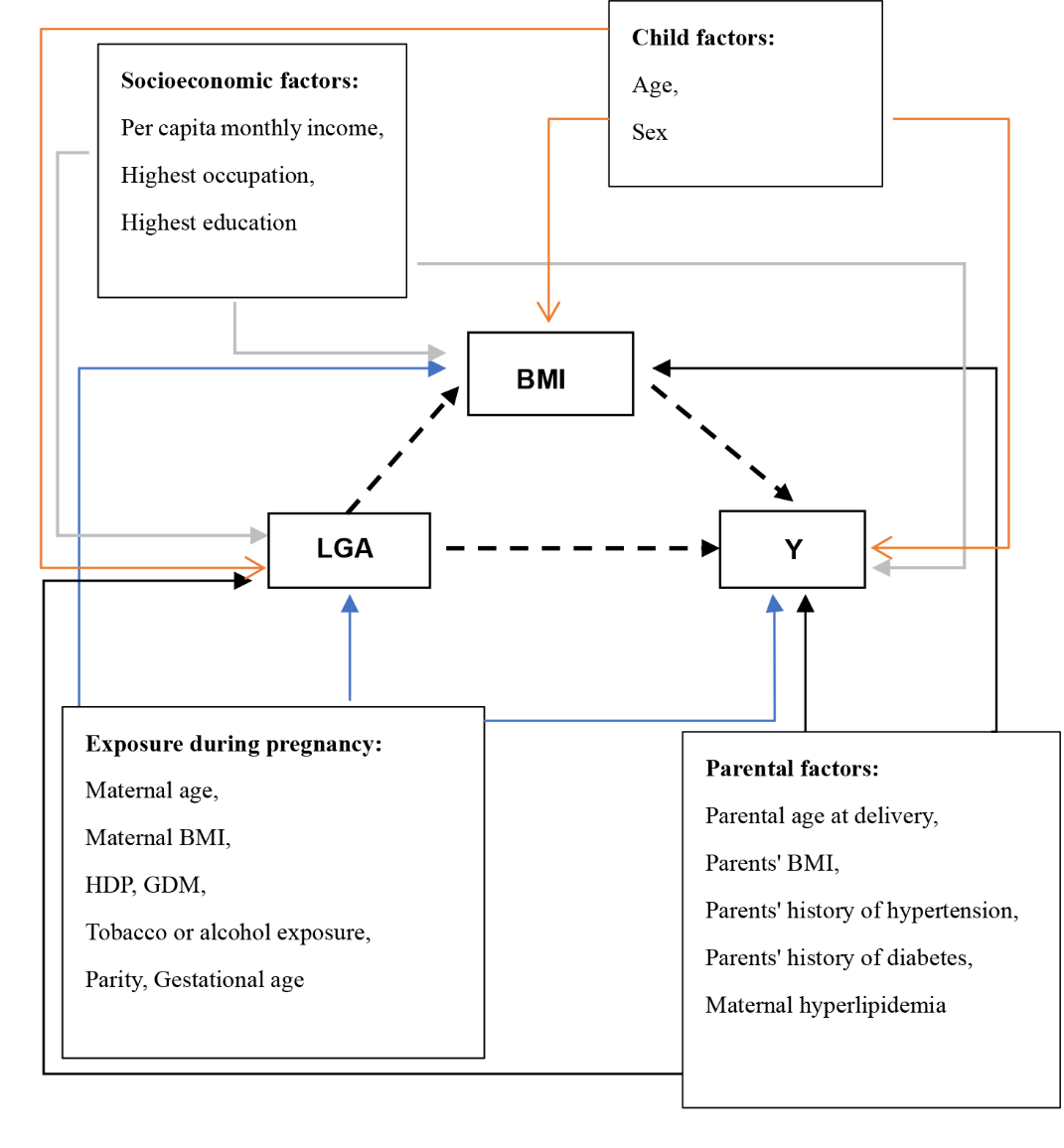
**

**Supplemental Figure 2:** Direct Acyclic Graph for the Models.

Models of systolic blood pressure, diastolic blood pressure, fasting blood glucose, fasting insulin, HOMA-IR were estimated. The dotted lines present the research question and the full lines the covariates adjusted for.

Abbreviations: LGA, large for gestational age; BMI, body mass index; GDM, gestational diabetes mellitus; HDP, hypertensive disorders in pregnancy.
