# Supplementary material for: Association between being large for gestational age and cardiovascular metabolic health in children conceived from assisted reproductive technology: a prospective cohort study
Source: BMC Med. 2024 May 20;22:203. doi: 10.1186/s12916-024-03419-7 (PMC11104001; doi:10.1186/s12916-024-03419-7)
Supplement: Supplementary file 3 — Additional file 3: Tab. S1. Children Characteristics, Parental Characteristics, and Family Socioeconomic Status in Included and Non-included Singleton Children Conceived from ART. [file 12916_2024_3419_MOESM3_ESM.docx]

**Supplementary Table 1:** Children Characteristics, Parental Characteristics, and Family Socioeconomic Status in Included and Non-included Singleton Children Conceived from ART.

|  | Non-included | Included | p value |
| --- | --- | --- | --- |
| **Child characteristics at birth** |  |  |  |
| Children, n | 15210 | 14048 |  |
| Female, n (%) | 7084 (46.6%) | 6752 (48.1%) | <0.001 |
| Length, cm | 50.21±2.25 | 50.33±1.85 | <0.001 |
| Birth weight, g | 3385.55±558.58 | 3473.65±485.67 | <0.001 |
| Gestational age, w | 39.09±1.77 | 39.06±1.53 | 0.098 |
| **Maternal characteristics** |  |  |  |
| Maternal age at delivery, y | 32.32±4.31 | 31.57±4.34 | <0.001 |
| Maternal pre-pregnancy BMI | 23.43±3.64 | 23.28±3.55 | <0.001 |
| Maternal height, cm | 161.90±5.27 | 161.73±4.98 | 0.005 |
| Gestational diabetes mellitus, n (%) | 1156 (7.6%) | 1046 (7.4%) | <0.001 |
| Hypertensive disorders in pregnancy, n (%) | 780 (5.1%) | 654 (4.7%) | <0.001 |
| Parity, n (%) |  |  | <0.001 |
| First born | 10176 (66.9%) | 11273(80.2%) |  |
| Second born | 3264 (21.5%) | 2664(19.0%) |  |
| Third born or later | 106 (0.7%) | 103 (0.7%) |  |
| **Paternal characteristics** |  |  |  |
| Paternal age, y | 33.05±4.91 | 32.38±4.90 | <0.001 |
| Paternal height, cm | 174.03±5.95 | 173.74±5.80 |  |
| Paternal BMI, kg/m^2^ | 25.90±4.13 | 25.70±4.03 |  |
| **Family socioeconomic status** |  |  |  |
| Per capita monthly income, n (%) |  |  | <0.001 |
| ＜3000 yuan | 3480 (22.9%) | 4947 (35.2%) |  |
| 3000~4999 yuan | 4978 (32.7%) | 5636 (40.1%) |  |
| ≥5000 yuan | 3080 (20.2%) | 3048 (21.7%) |  |
| Highest occupation |  |  | <0.001 |
| Student or unemployed | 1235 (8.1%) | 213 (1.5%) |  |
| Physical labor | 8389 (55.2%) | 9374 (66.7%) |  |
| Mental labor | 5567 (36.6%) | 4461 (31.8%) |  |
| Highest education |  |  | <0.001 |
| *High school or below | 8047 (52.9%) | 7844 (55.8%) |  |
| **University or above | 7145 (47.0%) | 6204 (44.2%) |  |

Data presented as mean ± SD for continuous variables and n (%) for categorical variables.

*High school or below: illiteracy, primary school, junior high school, and high school.

Abbreviations: BMI: body mass index.

**University or above: junior college, undergraduate, master's, and doctoral students.
