# Supplementary material for: Association between being large for gestational age and cardiovascular metabolic health in children conceived from assisted reproductive technology: a prospective cohort study
Source: BMC Med. 2024 May 20;22:203. doi: 10.1186/s12916-024-03419-7 (PMC11104001; doi:10.1186/s12916-024-03419-7)
Supplement: Supplementary file 4 — Additional file 4: Tab. S2. Effect of LGA on Measures of Childhood Anthropometric Data and Metabolisms. [file 12916_2024_3419_MOESM4_ESM.docx]

|  | Unadjusted | Model 1 | Model 2 | Model 3 | Model 4 |
| --- | --- | --- | --- | --- | --- |
| BMI, kg/m^2^ | **0.58** | **0.60** | **0.45** | **0.45** | 0.48 |
|  | **(0.51, 0.65)** | **(0.53,** **0.67)** | **(0.38,** **0.52)** | **(0.38, 0.52)** | (0.41, 0.55) |
| BMI z-score | **0.41** | **0.42** | **0.35** | **0.34** | 0.34 |
|  | **(0.37,0.46)** | **(0.37, 0.46)** | **(0.31,** **0.39)** | **(0.29,** **0.38)** | (0.30,0.38) |
| *Height, cm | **1.50** | **1.27** | **2.48** | **1.44** | 1.09 |
|  | **(0.92, 2.09)** | **(1.13, 1.42)** | **(1.90, 3.07)** | **(0.86, 2.03)** | (0.95, 1.24) |
| *Height z-score | **0.45** | **0.46** | **0.45** | **0.39** | 0.39 |
|  | **(0.42,** **0.49)** | **(0.42,** **0.49)** | **(0.41, 0.49)** | **(0.35,** **0.42)** | (0.36,0.43) |
| SBP, mmHg | **0.71** | **0.69** | **0.78** | **0.75** | 0.60 |
|  | **(0.29,** **1.13)** | **(0.34, 1.04)** | **(0.35, 1.20)** | **(0.32, 1.17)** | (0.25, 0.95) |
| SBP z-score | -0.01 | -0.01 | -0.02 | -0.01 | -0.01 |
|  | (-0.05, 0.03) | (-0.05, 0.02) | (-0.05, 0.02) | (-0.05, 0.02) | (-0.04, 0.03) |
| DBP, mmHg | **0.95** | **0.94** | **1.15** | **1.09** | 1.01 |
|  | **(0.51, 1.40)** | **(0.56,** **1.32)** | **(0.69, 1.60)** | **(0.64, 1.55)** | (0.61, 1.40) |
| DBP z-score | 0.03 | **0.04** | 0.03 | 0.03 | 0.05 |
|  | (0, 0.07) | **(0, 0.07)** | (0, 0.07) | (0, 0.07) | (0.02, 0.09) |
| FBG, mmol/L | **0.04** | **0.04** | **0.05** | **0.04** | 0.04 |
|  | **(0.02,0.07)** | **(0.02, 0.07)** | **(0.02, 0.07)** | **(0.02,0.07)** | (0.02,0.07) |
| FIN, mIU/L | **0.46** | **0.42** | **0.39** | **0.36** | 0.25 |
|  | **(0.22,** **0.71)** | **(0.20, 0.64)** | **(0.14, 0.64)** | **(0.11, 0.61)** | (0.03, 0.48) |
| HOMA-IR | **0.13** | **0.12** | **0.11** | **0.10** | 0.08 |
|  | **(0.06,** **0.19)** | **(0.06, 0.17)** | **(0.05, 0.17)** | **(0.04, 0.16)** | (0.02, 0.13) |
| TC, mmol/L | 0.03 | 0.03 | 0.03 | 0.03 | 0.04 |
|  | (-0.02, 0.07) | (-0.01, 0.07) | (-0.01, 0.08) | (-0.01, 0.08) | ( -0.01,0.08) |
| TG, mmol/L | 0 | 0 | 0 | 0 | 0 |
|  | (-0.02, 0.02) | (-0.02, 0.02) | (-0.02, 0.02) | (-0.02, 0.02) | (-0.02, 0.02) |
| LDL, mmol/L | 0.03 | 0.03 | 0.03 | 0.03 | 0.04 |
|  | (-0.01,0.06) | (-0.01, 0.07) | (0, 0.07) | (-0.01, 0.07) | (0, 0.08) |
| HDL, mmol/L | 0 | 0 | 0 | 0 | 0 |
|  | (-0.02,0.02) | (-0.02, 0.02) | (-0.02, 0.02) | (-0.02, 0.02) | (-0.02, 0.02) |
| BMI z-score >2 | **1.78** | **1.79** | **1.59** | **1.57** | 1.59 |
|  | **(1.63, 1.94)** | **(1.28,** **2.52)** | **(1.46,** **1.74)** | **(1.44,** **1.71)** | (1.46, 1.74) |
| **BP≥95th | 1.14 | 1.14 | 1.12 | 1.11 | 1.12 |
| percentile | (0.99, 1.31) | (0.99, 1.31) | (0.97, 1.29) | (0.96, 1.28) | (0.97, 1.29) |
| FBG≥5.6mmol/L | **1.35** | **1.37** | **1.35** | 1.33 | 1.35 |
|  | **(1.07, 1.70)** | **(1.07, 1.74)** | **(1.06, 1.71)** | (1.05, 1.69) | (1.05, 1.74) |
| HOMA-IR≥ | **1.66** | **1.79** | **1.52** | **1.50** | 1.58 |
| 95th percentile | **(1.33, 2.08)** | **(1.38,** **2.33)** | **(1.22,** **1.90)** | **(1.20, 1.87)** | (1.22, 2.06) |
| TC≥5.17mmol/L | 0.96 | 0.97 | 0.98 | 0.95 | 0.97 |
|  | (0.77, 1.20) | (0.77, 1.20) | (0.77, 1.23) | (0.76, 1.20) | (0.77, 1.22) |
| TG≥1.12mmol/L | 1.04 | 1.03 | 1.03 | 1.02 | 1.01 |
|  | (0.88, 1.23) | (0.87, 1.23) | (0.86, 1.22) | (0.86, 1.21) | (0.85, 1.21) |
| LDL≥3.36mmol/L | 1.10 | 1.10 | 1.10 | 1.09 | 1.11 |
|  | (0.88, 1.37) | (0.88, 1.37) | (0.88, 1.38) | (0.87, 1.36) | (0.89, 1.39) |
| HDL≤1.03mmol/L | 0.95 | 0.95 | 0.92 | 0.91 | 0.92 |
|  | (0.78, 1.15) | (0.78, 1.14) | (0.75, 1.12) | (0.74, 1.12) | (0.75, 1.12) |

**Supplementary Table 2:** Effect of LGA on Measures of Childhood Anthropometric Data and Metabolisms.

Data presented as their coefficients or OR and 95% CIs.

Model 1: adjusted for children's sex and age**.**

Model 2: adjusted for parity, gestational age, maternal age at delivery, HDP, GDM, maternal pre-pregnancy BMI, maternal tobacco and alcohol exposure during pregnancy

Model 3: adjusted for parents' BMI, history of hypertension, diabetes, maternal hyperlipidemia.

* Model 3: adjusted for parents' height, history of hypertension, diabetes, maternal hyperlipidemia.

Model 4: adjusted for Model 1+ Model 2+ Model 3+ socioeconomic factors

** According to the American Academy of Pediatrics diagnostic criteria for hypertension, only children ≥ 3 years old are included.

Bolded variables indicate statistical significance (q≤0.05)

Abbreviations: BMI, body mass index; SBP, systolic blood pressure; DBP, diastolic blood pressure; FBG, fasting blood glucose; FIN, fasting insulin; HOMA-IR, homeostatic model assessment for insulin resistance; LDL, low-density lipoprotein, HDL, high-density lipoprotein
