# Supplementary material for: Association between being large for gestational age and cardiovascular metabolic health in children conceived from assisted reproductive technology: a prospective cohort study
Source: BMC Med. 2024 May 20;22:203. doi: 10.1186/s12916-024-03419-7 (PMC11104001; doi:10.1186/s12916-024-03419-7)
Supplement: Supplementary file 6 — Additional file 6: Fig. S3. Visualization of BMI Changes with Age. [file 12916_2024_3419_MOESM6_ESM.docx]

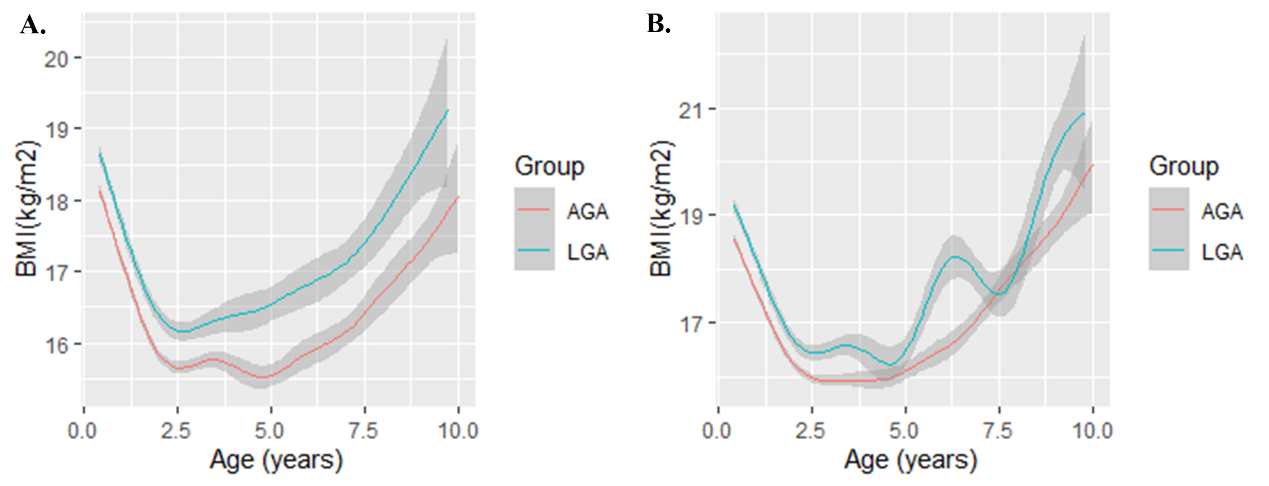


**Supplementary Figure 3:** Visualization of BMI Changes with Age

A: For female participants. B: For male participants

Abbreviation: BMI, body mass index; LGA, large for gestational age; AGA, appropriate for gestational age
