# Supplementary material for: Association between being large for gestational age and cardiovascular metabolic health in children conceived from assisted reproductive technology: a prospective cohort study
Source: BMC Med. 2024 May 20;22:203. doi: 10.1186/s12916-024-03419-7 (PMC11104001; doi:10.1186/s12916-024-03419-7)
Supplement: Supplementary file 7 — Additional file 7: Tab. S4. Measures of Anthropometry, Metabolic Markers of Offspring Born AGA, or LGA which Stratified by whether Embryos are Frozen. [file 12916_2024_3419_MOESM7_ESM.docx]

**Supplementary Table 4**: Measures of Anthropometry, Metabolic Markers of Offspring Born AGA, or LGA which Stratified by Whether Embryos are Frozen.

|  | **Fresh embryo transfer** | | | | | | Frozen embryo transfer | | | | | |
| --- | --- | --- | --- | --- | --- | --- | --- | --- | --- | --- | --- | --- |
|  | AGA | LGA | Unadjusted | | Adjusted | | AGA | LGA | Unadjusted | | Adjusted | |
|  |  |  | SE | q value | SE | q value |  |  | SE | q value | SE | q value |
| **Anthropometric characteristics** |  |  |  |  |  |  |  |  |  |  |  |  |
| n | 6347 | 2424 |  |  |  |  | 6957 | 3354 |  |  |  |  |
| Age, y | 2.52±2.04 | 2.64±2.12 |  |  |  |  | 1.75±1.57 | 1.82±1.62 |  |  |  |  |
| BMI, kg/m^2^ | 16.72±2.02 | 17.19±2.21 | **0.06** | **<0.001** | **0.06** | **<0.001** | 17.06±1.99 | 17.67±2.09 | **0.05** | **<0.001** | **0.05** | <0.001 |
| BMI z-score | 0.45±1.23 | 0.78±1.33 | **0.04** | **<0.001** | **0.04** | **<0.001** | 0.50±1.22 | 0.95±1.28 | **0.03** | **<0.001** | **0.03** | <0.001 |
| Height, cm | 90.15±18.15 | 92.44±18.86 | **0.53** | **<0.001** | **1.09** | **<0.001** | 83.04±15.16 | 85.03±15.67 | **0.38** | **<0.001** | **0.10** | <0.001 |
| Height z-score | 0.61±1.07 | 1.05±1.04 | **0.03** | **<0.001** | **0.03** | **<0.001** | 0.68±1.05 | 1.15±1.07 | **0.03** | **<0.001** | **0.03** | <0.001 |
| **BP** |  |  |  |  |  |  |  |  |  |  |  |  |
| n | 3259 | 1315 |  |  |  |  | 2374 | 1239 |  |  |  |  |
| Age, y | 3.93±1.89 | 4.00±1.94 |  |  |  |  | 3.38±1.63 | 3.39±1.65 |  |  |  |  |
| SBP, mmHg | 94.20±9.33 | 94.87±9.72 | 0.34 | 0.080 | 0.28 | 0.153 | 92.43±8.29 | 93.34±8.82 | **0.32** | **0.020** | **0.26** | 0.013 |
| SBP z-score | 0.04±0.83 | 0.02±0.80 | 0.03 | 0.601 | 0.03 | 0.501 | -0.04±0.68 | -0.01±0.72 | 0.03 | 0.483 | 0.02 | 0.845 |
| DBP, mmHg | 57.86±9.88 | 59.06±10.03 | **0.36** | **0.003** | **0.32** | **<0.001** | 55.21±8.46 | 56.34±9.26 | **0.33** | **0.005** | **0.28** | 0.001 |
| DBP z-score | 0.62±0.81 | 0.68±0.82 | 0.03 | 0.071 | **0.03** | **0.033** | 0.53±0.67 | 0.59±0.73 | **0.02** | **0.023** | **0.02** | 0.024 |
| **Metabolic characteristics,** |  |  |  |  |  |  |  |  |  |  |  |  |
| n | 2018 | 821 |  |  |  |  | 1319 | 681 |  |  |  |  |
| Age, y | 4.88±1.84 | 4.96±1.87 |  |  |  |  | 4.29±1.69 | 4.32±1.69 |  |  |  |  |
| FBG, mmol/L | 4.95±0.43 | 4.99±0.44 | 0.02 | 0.080 | 0.02 | 0.245 | 4.90±0.43 | 4.96±0.43 | **0.02** | **0.020** | **0.02** | 0.017 |
| FIN, mIU/L | 5.23±3.85 | 5.88±5.17 | **0.19** | **0.005** | 0.17 | 0.073 | 4.96±4.09 | 5.37±4.30 | 0.20 | 0.074 | 0.18 | 0.390 |
| HOMA-IR | 1.18±0.93 | 1.35±1.35 | **0.05** | **0.003** | **0.04** | **0.048** | 1.11±0.96 | 1.23±1.05 | **0.05** | **0.035** | 0.04 | 0.274 |
| TC, mmol/L | 4.05±0.73 | 4.08±0.69 | 0.03 | 0.413 | 0.03 | 0.255 | 4.02±0.69 | 4.08±0.72 | 0.04 | 0.450 | 0.04 | 0.390 |
| TG, mmol/L | 0.75±0.33 | 0.75±0.29 | 0.01 | 0.960 | 0.01 | 0.917 | 0.76±0.34 | 0.75±0.31 | 0.02 | 0.770 | 0.02 | 0.440 |
| LDL, mmol/L | 2.43±0.61 | 2.46±0.58 | 0.03 | 0.382 | 0.03 | 0.169 | 2.39±0.57 | 2.44±0.60 | 0.03 | 0.450 | 0.03 | 0.382 |
| HDL, mmol/L | 1.41±0.30 | 1.42±0.29 | 0.01 | 0.601 | 0.01 | 0.797 | 1.41±0.30 | 1.42±0.30 | 0.02 | 0.770 | 0.02 | 0.712 |

Data presented as mean ± SD for continuous variables and n (%) for categorical variables.

Bolded variables indicate statistical significance (q≤0.05).

Adjusted: Adjusted for children's age and sex, parity, gestational age, parental age at delivery, HDP, GDM, maternal tobacco and alcohol exposure during pregnancy, parents' BMI,

parents' history of hypertension and diabetes, maternal hyperlipidemia, socioeconomic factors.

*Adjusted: Adjusted for children's age and sex, parity, gestational age, parental age at delivery, HDP, GDM, maternal tobacco and alcohol exposure during pregnancy, parents' height,

parents' history of hypertension and diabetes, maternal hyperlipidemia, socioeconomic factors.

Abbreviations: SE, standard error; BMI, body mass index; BP, blood pressure; SBP, systolic blood pressure; DBP, diastolic blood pressure; FBG, fasting blood glucose; FIN,

fasting insulin; HOMA-IR, homeostatic model assessment for insulin resistance; TC, total cholesterol; TG, Triglyceride; LDL, low-density lipoprotein; HDL, high-density lipoprotein.
