# Supplementary material for: Association between being large for gestational age and cardiovascular metabolic health in children conceived from assisted reproductive technology: a prospective cohort study
Source: BMC Med. 2024 May 20;22:203. doi: 10.1186/s12916-024-03419-7 (PMC11104001; doi:10.1186/s12916-024-03419-7)
Supplement: Supplementary file 9 — Additional file 9: Tab. S6. Number of Children at Each Follow-up Stage. [file 12916_2024_3419_MOESM9_ESM.docx]

**Supplementary Table 6:** Number of Children at Each Follow-up Stage.

|  | Age | Number |
| --- | --- | --- |
| Neonatal period | 0-42d | 14048 |
| Infancy | 42d-0.9y | 7058 |
| Toddler's age | 1-2.9y | 9915 |
| Preschooler | 3-5.9y | 4060 |
| School age children | 6-9.9y | 1355 |
